# Determining the Optimal Heparin Binding Domain Distance in VEGF_165_ Using Umbrella Sampling Simulations for Optimal Dimeric Aptamer Design

**DOI:** 10.3390/ijms27020712

**Published:** 2026-01-10

**Authors:** Jung Seok Lee, Yeon Ju Go, Young Min Rhee

**Affiliations:** Department of Chemistry, Korea Advanced Institute of Science and Technology (KAIST), Daejeon 34141, Republic of Korea; jungseok362@kaist.ac.kr (J.S.L.); koyj2018@kaist.ac.kr (Y.J.G.)

**Keywords:** VEGF, umbrella sampling, dimeric aptamer, molecular dynamics simulation

## Abstract

Vascular endothelial growth factor 165 (VEGF_165_) stands out as a pivotal isoform of the VEGF-A protein and is critically involved in various angiogenesis-related diseases. Consequently, it has emerged as a promising target for diagnosing and treating such conditions. Structurally, VEGF_165_ forms a homodimer, and each of its constituting monomers comprises a receptor-binding domain (RBD) and a heparin-binding domain (HBD). These two domains are linked by a flexible linker, and thus the overall structure of VEGF_165_ remains incompletely understood. Aptamers are known as potent drugs that interact with VEGF_165_, and dimeric aptamers that can simultaneously interact with two distant domains are frequently adopted to improve the potency. However, designing such aptamer dimers faces challenges in regard to determining the appropriate length of the linker connecting the two aptamer fragments. To gain insight into this distance information, we here employ biased molecular dynamics (MD) simulations with the umbrella sampling method, with the distance between the two HBDs serving as a reaction coordinate. Our simulations reveal an overall preference for compact conformations with HBD-HBD distances below 3 nm, with the minimum of the potential of mean force located at 1.1 nm. We find that VEGF_165_ with the optimal HBD-HBD distance forms hydrogen bonds with its receptor VEGFR-2 that well match experimentally known key hydrogen bonds. We then try to computationally design aptamer homodimers consisting of two del5-1 aptamers connected by various linker lengths to target VEGF_165_. Collectively, our findings may provide quantitative guidelines for rationally designing high-affinity aptamers for targeting VEGF_165_.

## 1. Introduction

Vascular endothelial growth factor (VEGF) regulates physiological vasculogenesis and angiogenesis by binding to and activating the VEGF receptor (VEGFR) family [1,2,3,4,5,6]. VEGF has been implicated in pathological angiogenic diseases and has made itself a key target for diagnosing and treating such conditions [2,3,7,8,9,10,11]. There are five different types of human VEGF, among which VEGF-A, often referred to as just VEGF, is known to be most important with its own several isoforms [11]. VEGF_165_ is the most prevalent one and it consists of two monomers forming a homodimeric structure. Each monomer contains a receptor binding domain (RBD) and a heparin binding domain (HBD) and they are connected by a flexible linker [11,12]. While the structures of both binding domains have been characterized experimentally, the complete VEGF_165_ structure remains elusive due to its flexible linker. Thus, efforts have been made to gain insights on the full structure and to understand the dynamics of VEGF_165_ through computational methods [12,13,14]. Some studies suggested that the two HBDs exhibit significant scissoring motion due to the flexible linker [13,14]. However, a clear understanding of the optimal inter-HBD distance and its distribution is still lacking, posing a significant challenge in drug development. Indeed, the development of aptamer dimers with enhanced avidity toward VEGF_165_ through simultaneous targeting of two HBDs or both RBD and HBD [15,16,17,18,19,20] will greatly benefit from accurate inter-domain distance information [15,16]. Namely, this information may enable the rational determination of optimal linker length for the aptamer dimer design [16,17].

To address this challenge, we in this work employ biased molecular dynamics (MD) simulations with the umbrella sampling method (USM) [21] by considering the distance between the two HBDs as the reaction coordinate (RC). Our main goal is to determine whether the free energy represented by the potential of mean force (PMF) with respect to the reaction coordinate displays abrupt or gradual changes, thereby uncovering the extent of flexibility in VEGF_165_ linker and identifying whether multiple stable regions exist. We will show that the PMF of VEGF_165_ exhibits an inverted-bell shape with distinct minima, corresponding to optimal distance between the two HBDs. This suggests that a single well-designed aptamer homodimer with an optimal HBD-HBD distance can effectively target VEGF_165_.

To determine whether VEGF_165_ binding to its typical receptor VEGFR-2 requires adjustments in the distance between the two HBDs of VEGF_165_, we conduct additional MD simulations of VEGFR-2/VEGF_165_ complexes with varying HBD distances. Analysis of hydrogen bond (H-bond) formation between VEGF_165_ and VEGFR-2 reveals that VEGF_165_ with optimal HBD distances, as determined by the PMF analysis, effectively binds to the receptor and engages in robust interactions. We will show that the residues involved in the hydrogen bond are consistent with those identified in experiments, underscoring the reliability of the optimal HBD distances determined through our MD simulations using USM. We will then try to computationally design aptamer homodimers targeting the two HBDs of these VEGF_165_ structures by adopting two del5-1 aptamers connected by thymine nucleotide linkers of various lengths (del5-1:del5-1). Through docking calculations, we determine the binding conformations between VEGF_165_ and the aptamer homodimer. Our analysis provides critical considerations for designing aptamer homodimers that can effectively bind to VEGF_165_.

## 2. Results and Discussion

### 2.1. PMF Along the Reaction Coordinate of VEGF165

**Figure 1:**
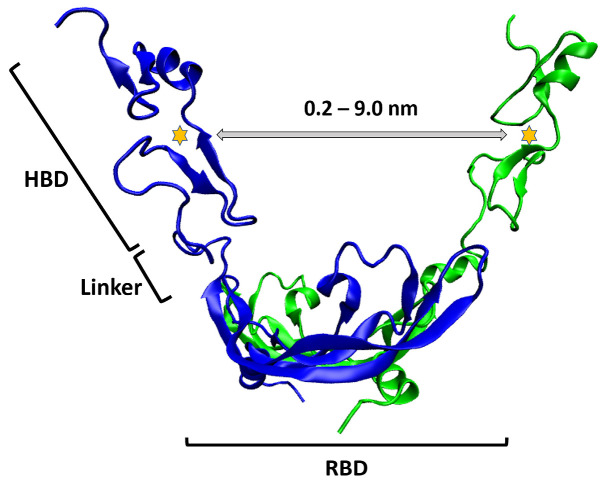
Schematic depiction of the RC for VEGF_165_. The two VEGF_165_ monomers are illustrated in blue and green, while the yellow stars represent the COMs of HBD, signifying the inter-monomer COM distance between the two HBDs. This distance is defined as the RC and is employed in the PMF simulations.

Previous studies stressed the importance of accounting for both large- and small-scale motions of VEGF_165_ for reliably elucidating its interaction with aptamers [13,14]. Additionally, these studies proposed that the large-scale motion of VEGF_165_ involves a scissoring motion, and computationally explored a range of HBD-HBD distances to model this effect [13,14]. However, to reduce the computational cost, the anisotropic network model (ANM) was employed toward ascertaining the large-scale motion, thereby sacrificing some accuracy compared to MD simulations with detailed interactions. Hence, in this work, we report on MD simulations using umbrella sampling to investigate the flexibility of VEGF_165_ across a wide range of HBD-HBD distances. Our aim is to determine whether the dimer exhibits a scissoring motion around one stable conformation with the HBDs oscillating widely, or possesses multiple stable regions with local free energy minima. The RC was defined as the distance between the centers of mass (COMs) of the two HBDs of VEGF_165_ (Figure 1), explored over a range of 0.2 to 9.0 nm. Choosing this range was based on insights derived from the earlier ANM analyses [13,14] and another computational study suggesting a sandwich-like binding configuration of VEGF_165_ to VEGFR-2 [12].

**Figure 2:**
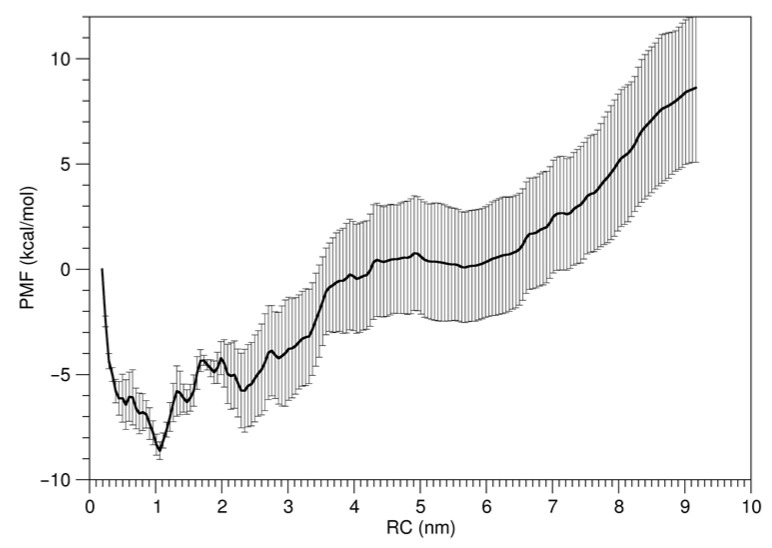
PMF as a function of the distance between the two HBD units of VEGF_165_. Error bar represents standard deviation of dataset of last 100 ns of the production phase of umbrella sampling trajectories divided into five independent 20 ns blocks.

To explore the free energy landscape along the reaction coordinate (RC) defined by the center-of-mass (COM) distance between the two HBDs, the potential of mean force (PMF) was calculated using umbrella sampling (Figure 2). The reliability of the sampling was validated by confirming the significant overlap between adjacent windows (Figure S1). The individual PMF profiles from the five 20 ns blocks used to calculate the error bars in Figure 2 are provided in Figure S2. Through the analysis of the PMF profile shown in Figure 2, we can observe that the free energy reaches a minimum when the distance between two HBDs of VEGF_165_ is 1.1 nm, with another local minimum at 2.3 nm appearing as the second favorable distance. A significant free energy change of 17.0 kcal/mol is also observed as the distance decreases from 9.0 nm to 1.1 nm. Namely, this PMF profile indicates a progressive and nearly monotonic reduction in the distance between the HBDs until stabilization around at 1.1 nm, and at this distance VEGF_165_ adopts a sandwich-like conformation. Our results show the dominant large-scale dynamics is a unidirectional transition toward this stable and compact conformation, somewhat differently from the scissoring motion suggested earlier with ANM [13,14].

**Figure 3:**
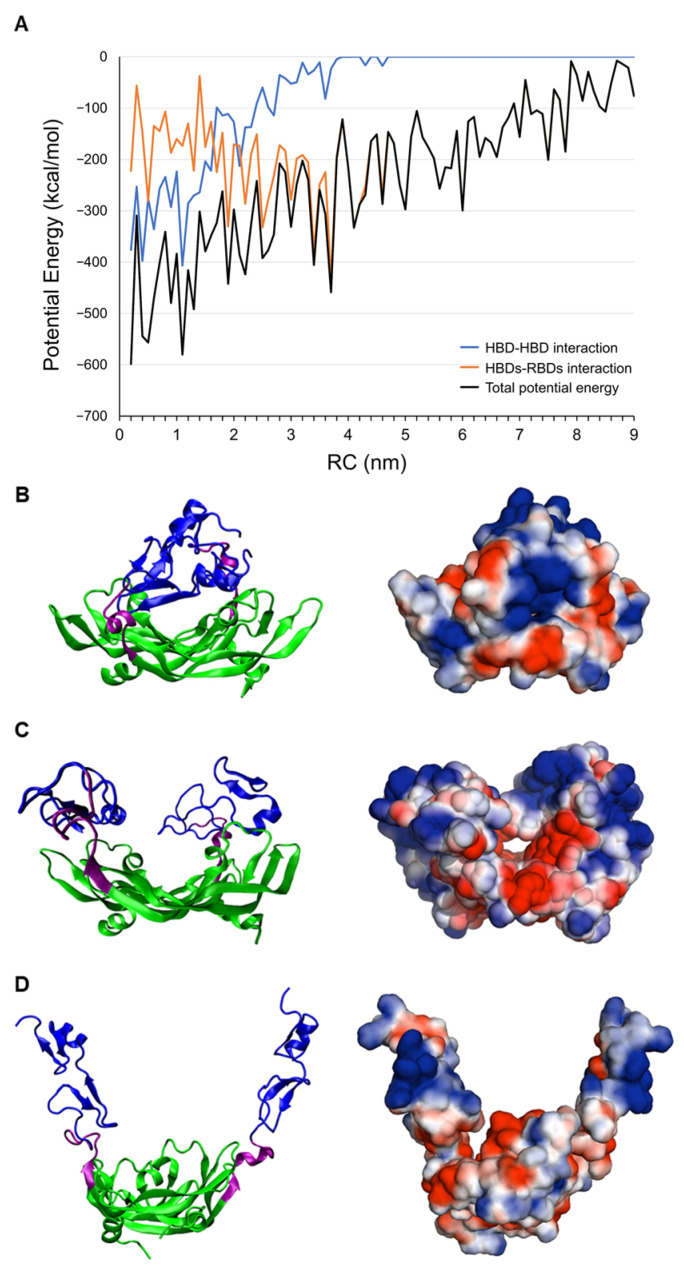
(**A**) Profiles of inter-domain interaction energies along the reaction coordinate (RC): HBD–HBD interaction energy (blue), HBD–RBD interaction energy (orange), and their sum (black). (**B**–**D**) Solvent-accessible surface electrostatic potentials of VEGF_165_ at its varying structures with HBD-HBD distances of (**B**) 1.1 nm, (**C**) 3.5 nm, and (**D**) 9.0 nm. The conformations were taken from the last frames of the biased MD trajectories with the umbrella potentials centered at each given distance. The left panels show the structures with a coloring scheme of RBD: green, linker: purple, and HBD: blue. The right panels display the solvent-accessible surface electrostatic potential, with red indicating negative and blue indicating positive electrostatic potential, represented on a scale ranging from −1.5 to 1.5 *k*_B_*T*/*e* with *T* = 298 K.

To rationalize this stabilization, we calculated the interaction energy profiles between the HBD–HBD pair and the HBD–RBD pairs along the RC (Figure 3A). The results show that the HBD–HBD interaction diminishes to zero at RC > 4.0 nm, while the HBD–RBD interaction dominates the total potential in the range of 3.0–4.0 nm. This indicates that at large RCs, the HBDs are initially driven toward smaller RC values primarily by interactions with the RBDs; once they are sufficiently close, the HBD–HBD interaction begins to contribute to the overall stabilization. We also calculated the solvent-accessible surface electrostatic potential around VEGF_165_ using the APBS tool in PyMOL (version 1.9.0) [22], with atomic charges of proteins and aptamers generated by PDB2PQR serving as input for APBS. This revealed that the RBD region of VEGF_165_ generally exhibits a negative electrostatic potential, while the HBD region tends to exhibit a positive electrostatic potential (Figure 3B–D). When the two HBDs of VEGF_165_ forms a sandwich-like compact structure (observed at HBD–HBD distances below 3.0 nm), two HBDs are observed to be closely associated (Figure 3B). This tight electrostatic interaction becomes absent in extended VEGF_165_ conformations where the HBD–HBD distance exceeds 3.0 nm (Figure 3C,D). This will be the reason VEGF_165_ adopts a stable and relatively compact conformation rather than displaying wide structural fluctuations. When the trajectory simulations were extended for additional 50 ns starting from structures obtained at the end of the biased MD simulations but by turning off the biases, we observed a tendency for RC to decrease, particularly in trajectories that started from extended conformations (Figure S3). This structural insight indicates that aptamer dimer targeting both HBDs should maintain optimal separation distance to bind VEGF_165_ effectively. Furthermore, elucidating whether VEGF_165_ binds to VEGFR-2 directly from its thermodynamically stable conformation or needs to involve conformational rearrangement prior to binding VEGFR-2 may be an important issue for dimeric aptamer design. These will be the points of discussion in the following sections.

### 2.2. HBD-HBD Distance Change in VEGF_165_ upon VEGFR-2 Binding

**Figure 4:**
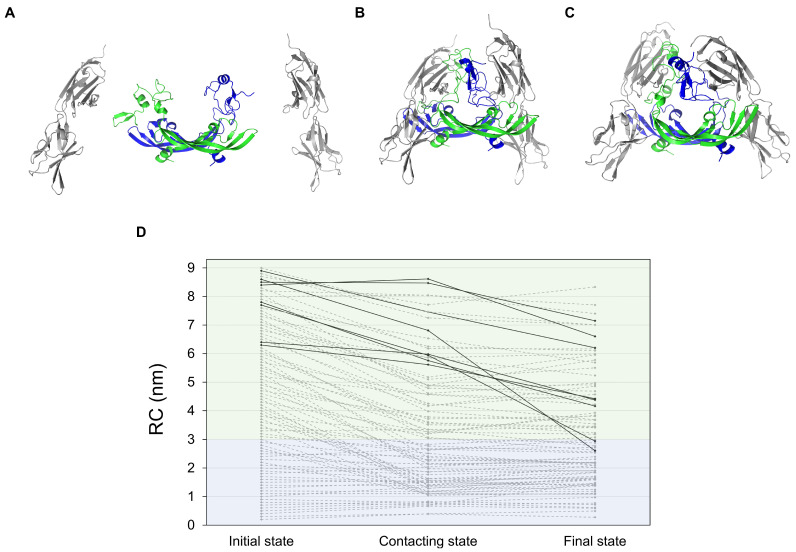
VEGFR-2/VEGF_165_ complex formation demonstrates preferential binding with compact VEGF_165_ conformation. (**A**) Initial state showing VEGFR-2 positioned 4.0 nm away from VEGF_165_ RBD COM for the case with the HBD-HBD distance at 4.5 nm. (**B**) Contacting state immediately after rigid-body translation of VEGFR-2 toward VEGF_165_ RBDs. (**C**) Final relaxed VEGFR-2/VEGF_165_ complex after 100 ns of an unrestrained MD simulation. (**D**) Changes in the HBD-HBD distance for all 89 trajectories across the three states. Gray dashed lines represent individual trajectories, while black solid lines highlight systems with RC changes of 1 nm or greater from the contacting to the final state. Notably, all highlighted systems show a decrease in RC during equilibration. Systems with compact initial conformations (RC < 3.0 nm, blue-shaded region) remain stable, whereas those with extended conformations (RC ≥ 3.0 nm, green-shaded region) tend to become more compact over time, exhibiting larger structural changes.

To investigate whether the binding of VEGF_165_ to VEGFR-2 requires adjustments in the HBD–HBD distance, we constructed 89 initial complex models. During the initial structural overlay onto the experimental template (PDB ID: 3V2A) [23], steric clashes occurred especially for conformations with HBD–HBD distances exceeding 3 nm. To circumvent this, the two VEGFR-2 units were temporarily translated 4 nm away from the RBD. This procedure created sufficient separation to eliminate any steric clashes (Figure 4A). Each VEGFR-2 was then pulled back along the same vector toward dimeric RBD COM until reaching their initial superimposed positions (Figure 4B). The generated 89 VEGFR-2/VEGF_165_ complexes then underwent MD simulations for 100 ns (Figure 4C).

Analyses with these procedures revealed distinct behaviors depending on the initial HBD-HBD distance. During the pulling simulations, complexes with compact conformations maintained their HBD-HBD distances, but the ones with extended conformations gradually changed the geometries into decreased HBD-HBD distances, indicating that the binding pocket formed by the two VEGFR-2 units preferentially accommodates VEGF_165_ in its compact form. This trend persisted in subsequent MD simulations. Namely, initially extended conformations transitioned toward the compact side, while the compacted VEGF_165_ generally remained compact without expanding beyond the level of thermal fluctuations during subsequent unrestrained simulations (Figure 4D), consistently with the PMF profile. Therefore, we can infer that the compact conformation is not only a thermodynamically stable state of VEGF_165_ but also the crucial conformation for productive binding to VEGFR-2. We also checked whether the initial pulling speed (0.01 nm ps^−1^) was too fast for some extended conformations to fully relax to compaction. For all trajectories initiated with RC ≥ 6.0 nm, which occasionally failed to compact at this speed (Figure 4), we conducted new pulling simulations at a ten times slower rate (0.001 nm ps^−1^). With this slower speed, all trajectories showed a clear tendency to compact (Figure S4), confirming that this compacting behavior is a genuine feature of the system and not an artifact of trapping induced by an artificial pulling speed.

### 2.3. Hydrogen Bond Formation Between VEGF_165_ and VEGFR-2

**Figure 5:**
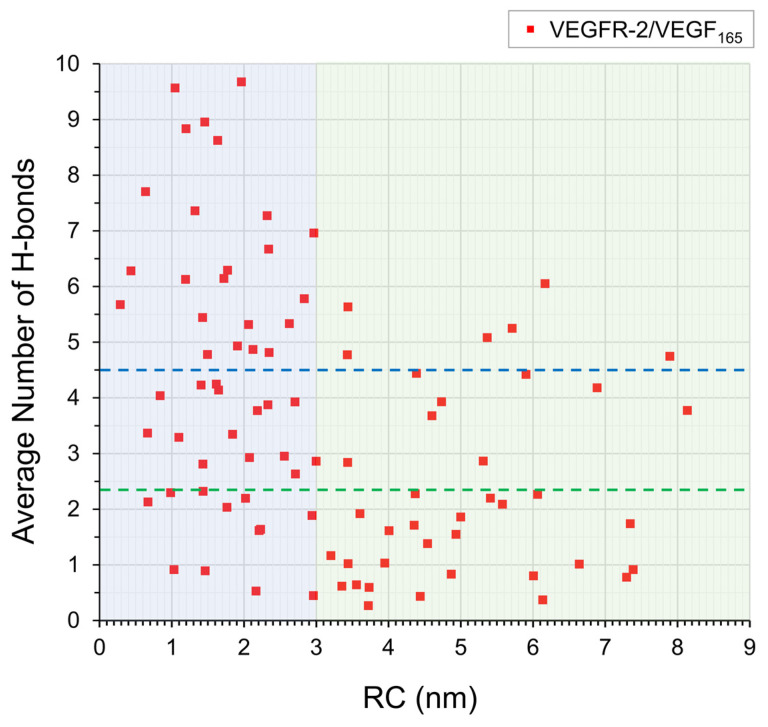
The average number of key H-bonds established between VEGFR-2 and VEGF_165_. The horizontal axis (RC) represents average distance between the two HBDs of VEGF_165_ over the 50–100 ns simulation windows. The blue-shaded region (RC < 3.0 nm) represents compact, sandwich-like structures and includes the two minima observed in the PMF profile. The green-shaded region (RC ≥ 3.0 nm) corresponds to extended conformations. Horizontal blue and green dotted lines represent the mean number of hydrogen bonds calculated within blue and green regions.

**Figure 6:**
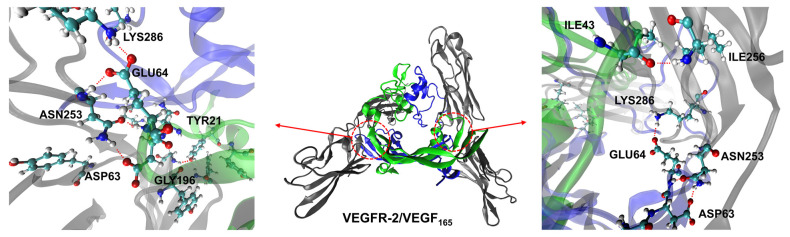
Illustration of the VEGFR-2/VEGF_165_ complex and its H-bonds formed between the two proteins. VEGFR-2 is depicted in silver, while monomers A and B of VEGF_165_ are shown in blue and green. On the left panel, hydrogen bonds between VEGFR-2 and the monomer A of VEGF_165_ are depicted along with relevant residues, while on the right panel the same information is shown for the monomer B of VEGF_165_. These H-bonds are represented by red dashed lines.

To validate the biological relevance of the compact conformation in our simulations, we next analyzed their ability to reproduce the 10 specific key hydrogen bonds observed in experimental studies of VEGFR-2/VEGF_165_ complex (Table S1) [23]. For this hydrogen bond analysis, snapshots were extracted at every 10 ps from the 50–100 ns windows of the 100 ns MD trajectories of the complexes. Because of the dimeric nature of the complex, we considered the possibilities of 20 hydrogen bonds in total. This analysis revealed that complexes with compact VEGF_165_ conformations formed an average of 4.40 hydrogen bonds, which is significantly higher than the average of 2.38 hydrogen bonds observed in complexes with extended conformation (Figure 5). Notably, the conformations exhibiting the highest hydrogen bond counts were mostly in the 1–2 nm range, encompassing the energy minimum identified within the explored PMF profile. The prevalence of hydrogen bonds in the compact conformation arises because an increased HBD-HBD distance leads to a higher VEGFR-2 root-mean-square deviation (RMSD), indicating significant structural distortion compared to the experimental structure (Figure S5). This implies that the thermodynamically most stable conformation also achieves an optimal binding geometry and demonstrates that the results of our biased MD simulations with the umbrella sampling are meaningful. To illustrate the formation of key H-bonds, in Figure 6, we depicted the structure of the VEGFR-2/VEGF_165_ complex obtained after 100 ns simulation with a final HBD distance of 1.1 nm. The ability of the compact VEGF_165_ conformations to form a robust interaction network with VEGFR-2 that reproduces key experimental hydrogen bonds provides compelling evidence that the low-energy basin identified in our PMF profile corresponds to the functionally relevant and optimal binding geometry.

### 2.4. Computationally Designing an Aptamer Homodimer Targeting VEGF_165_

**Figure 7:**
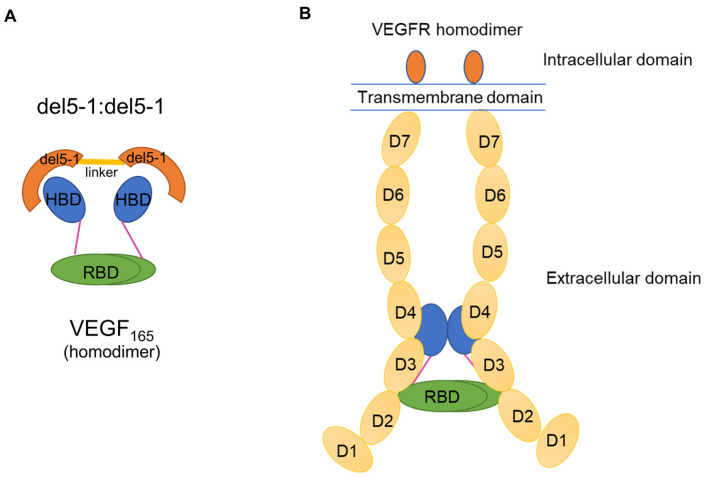
(**A**) Schematic representation of the proposed binding mode between the del5-1:del5-1 aptamer dimer and the VEGF_165_ homodimer. The orange ovals represent the two del5-1 aptamer domains connected by a flexible linker, which “encircle” the HBD of the VEGF_165_ by coulombic interaction. (**B**) Architectural overview of the native VEGF_165_/VEGFR homodimer complex on the cell membrane. This illustration highlights that del5-1:del5-1 aptamer dimer could potentially interfere with the interaction between the VEGF_165_ ligand and the VEGFR homodimer, thereby modulating or inhibiting the downstream signaling pathway.

Based on the optimal distance we discovered between the two HBDs of VEGF_165_, we aimed to provide insights for designing aptamer homodimer linkers that can induce simultaneous binding to both HBDs. It has been experimentally shown that creating an aptamer homodimer by linking two VEa5 mutants, del5-1, results in better binding affinity with VEGF_165_ in comparison with the monomeric aptamer [15,16]. The structural basis for this enhanced affinity and the potential inhibition mechanism are illustrated in Figure 7. Figure 7A depicts how the dimerized aptamer simultaneously captures both HBDs, while Figure 7B illustrates how this binding could potentially interfere with the native VEGF/VEGFR signaling assembly [4,5,14] on the cell membrane. However, due to the flexibility of the linker connecting the two del5-1 fragments, neither the overall structure of del5-1:del5-1 nor its binding structure with VEGF_165_ is known.

To gain an insight, we investigated whether varying the length of a thymine-only linker connecting the two del5-1 aptamers would affect their binding affinity to VEGF_165_ by performing docking calculations. We computationally constructed a total of 13 types of del5-1:del5-1 aptamers: one without a linker (0 dT), and others with linkers consisting of 1 to 10 thymine nucleotides (1–10 dTs), as well as linkers with 15 and 20 thymine nucleotides (15 and 20 dTs). Each del5-1:del5-1 underwent energy minimization and a 1500 ns unbiased MD simulation. From the window between 500 ns and 1500 ns, we obtained a total of 101 del5-1:del5-1 snapshots at 10 ns intervals.

**Figure 8:**
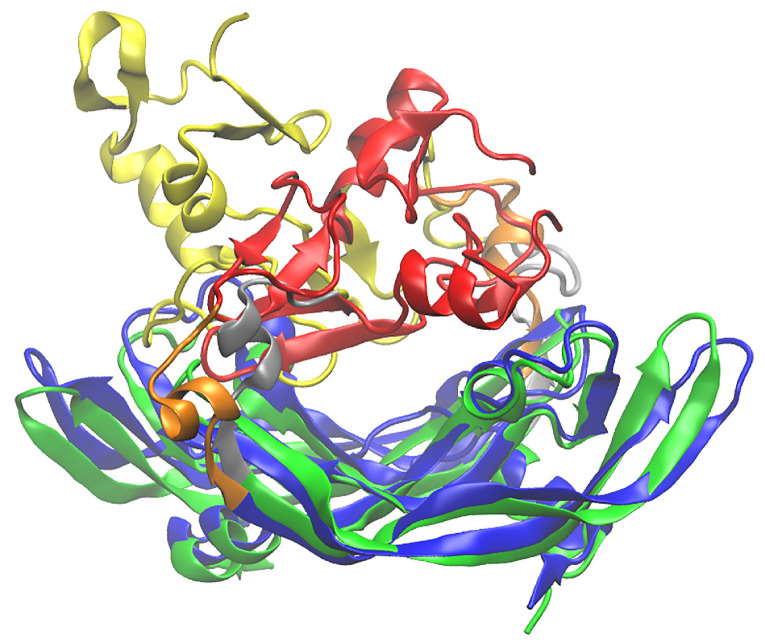
Illustration of the structural diversity of VEGF_165_. The widely different two conformations correspond to the final snapshots obtained from independent 200 ns biased MD simulations, both bearing the same HBD–HBD separation of 1.1 nm. One VEGF_165_ is depicted with its RBD part in blue, HBD in red, and linker in orange, while the other one is shown with its RBD part in green, HBD in yellow, and linker in grey.

**Figure 9:**
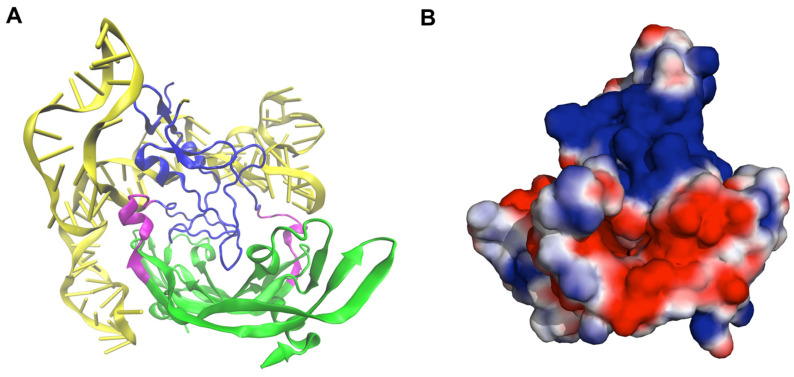
Structural and electrostatic characterization of the VEGF_165_/del5-1:del5-1 complex with a 0 dT linker. (**A**) Representative conformation of the complex with the highest docking score. The VEGF_165_ homodimer is depicted with the RBD in green, the HBD in blue, and the linker in magenta. The two del5-1 aptamers are highlighted in yellow. (**B**) Solvent-accessible surface electrostatic potential of VEGF_165_ for the same snapshot shown in (**A**). The surface is colored by the electrostatic potential, where red and blue indicate negative and positive regions, respectively, on a scale ranging from −1.0 to 1.0 *k*_B_*T*/*e* with *T* = 298 K. The DNA aptamer have been omitted to clearly illustrate the prominent positive surface charge concentrated on the HBD, which plays a critical role in aptamer recognition and binding.

The fact that the two HBD units prefer a separation of 1.1 nm distance does not necessarily mean that the overall VEGF_165_ conformation is rigid. To investigate how structurally diverse VEGF_165_ may be at the optimal separation, we conducted additional 10 independent 200 ns biased MD simulations of VEGF_165_ with different initial velocities, in addition to the two umbrella sampling trajectories already performed. These trajectories indeed yielded diverse VEGF_165_ structures (Figure 8). Subsequently, we obtained a total of 72 VEGF_165_ snapshots at 20 ns intervals from the 100–200 ns segments of these 12 MD trajectories. We then used these 72 VEGF_165_ and del5-1:del5-1 structures to obtain complex structures using rigid body docking with HDOCK [24]. We averaged the docking scores of VEGF_165_ and del5-1:del5-1 based on the type of linker used for del5-1:del5-1. While the linker with 20 dTs exhibited the highest docking score, no significant positive or negative correlation was observed between the linker length and the binding affinity (Table 1). However, upon analyzing the top ten complexes based on docking scores for each of the 13 different linker lengths, we observed that the distance between the COM of the two del5-1 aptamers in del5-1:del5-1 averaged between 4.5–5.6 nm regardless of the actual linker lengths. Detailed inspection on the complex structures using VMD [25] revealed that del5-1:del5-1 commonly binds in a manner that encircles the two HBDs (Figure 9A). As illustrated in the surface electrostatic potential map (Figure 9B), HBDs exhibit a highly concentrated positive surface potential, which effectively recruits and anchors the negatively charged phosphate backbones of the del5-1 aptamers through coulombic interactions. This indicates that, although determining the optimal length of the linker in an aptamer dimer is challenging due to its flexibility as shown in various studies [16,26], for del5-1:del5-1 targeting VEGF_165_, it is crucial to design del5-1:del5-1 such that the COM distance between the two del5-1 aptamer domains can become ~5 nm to effectively wrap the cluster of the two HBDs. Perhaps optimization is not even crucial as long as the linker is flexible enough, as the HBDs will display quite a well-defined distance as suggested by our PMF.

To validate the structural and thermodynamic stability of the predicted binding modes, we performed 100 ns MD simulations starting from the top five docking poses of all 13 linker systems, amounting to a total of 65 independent trajectories. The structural integrity was assessed by performing molecular mechanics Generalized Born surface area (MM-GBSA) free energy calculations. The comprehensive results are summarized in Table S2. Despite occasional dissociation observed in a few trajectories, we could consistently identify stable binding modes for every linker system. For these stable complexes, the COM distances between each del5-1 aptamer domain and the HBD of VEGF_165_ remained consistent with the initial docking configurations throughout the production phase. Furthermore, the MM-GBSA results demonstrate that these complexes possess favorable binding energies, providing further energetic evidence that these binding modes are thermodynamically stable in a solvated environment.

## 3. Materials and Methods

### 3.1. Modeling VEGF_165_

The complete experimental structure of homodimeric VEGF_165_ remains unknown. Nevertheless, the X-ray crystal structure of the homodimeric RBD (PDB ID: 2VPF) [27] and the NMR structure of the monomeric HBD (PDB ID: 1VGH) [28] were at our disposal. Thus, we manually established connections between each HBD and its corresponding RBD monomer using the sequence information, incorporating the interdomain linker sequence RPKKDRARQENP. Here, RPKKD and ARQENP respectively align with the X-ray crystal structure of RBD (PDB ID: 2VPF) and the NMR structure of HBD (PDB ID: 1VGH). The absent residue R110 was generated using Avogadro [29]. Through the application of VMD [25], we manually linked the individual components to complete the construction of the entire structure of homodimeric VEGF_165_. The overall fold of the constructed model is consistent with the prediction from AlphaFold 3 (see Figure S6 for a visual comparison) [30]. This was followed by stabilization through energy minimization and equilibration, whose details will be described in Section 3.5.

### 3.2. Modeling VEGFR-2/VEGF_165_

The X-ray crystal structure of the complex involving domains 2 and 3 (D23) of VEGFR-2 and VEGF-A corresponding to the homodimeric RBD region of VEGF_165_ is known (PDB ID: 3V2A) [23]. This experimental structure contains missing residues, and we placed these missing residues using the homology modeling method with SWISS-MODEL [31] toward completing the dimeric structure of the VEGFR-2 D23/VEGF_165_ (referred to as VEGFR-2/VEGF_165_ throughout this paper).

### 3.3. Modeling DNA Aptamer

To build the model structure of del5-1:del5-1 with a linker, we first built its secondary structure from the sequence using the Mfold web server [32] and then generated the 3D model via the 3dDNA web server [33]. In both steps, energetically the most stable structure among the predicted ones was selected. The sequence of del5-1 is 5′-ATACC AGTCT ATTCA ATTGG GCCCG TCCGT ATGGT GGGTG TGCTG GCCAG-3′. A total of 13 different linker lengths, namely 0, 1, 2, …, 10, 15, 20 thymines, were adopted to generate del5-1:del5-1 3D structures. For each structure model, we performed energy minimization and unbiased MD simulation over a 1500 ns duration. Detailed information on the MD simulation is described in Section 3.5.

### 3.4. Docking Simulations

All docking simulations were performed using HDOCK [24], a program that is widely applied for protein-RNA and protein-DNA docking studies [34,35]. It has demonstrated exceptional performance in the community-wide Critical Assessment of PRediction of Interactions (CAPRI) [36], operating as a rigid body docking tool where the target remains stationary while the ligand explores translational and rotational space in fixed increments. Rotational sampling was conducted at 15-degree intervals, and translational sampling, based on fast Fourier transform (FFT), used a step size of 0.12 nm [24]. From these sampling procedures, a shape-based pairwise scoring function was employed to assess binding modes. The top ten translations showing the best shape complementarity were further refined using an iterative knowledge-based scoring function, ensuring that optimal docking results were retained for each rotation [24].

### 3.5. MD Simulations

After generating each model structure, the system was solvated in TIP3P water [37]. The minimum distance between the solute and the box edge was set to 5 nm, and counter ions of sodium or chloride were added for charge neutralization. The AMBER ff19SB force field parameters for VEGF_165_ were adopted [38], and the topology file for simulation was generated using AmberTools22 [39]. This was followed by energy minimization with the steepest descent method [40] with convergence reached when the maximum force was below 1000 kJ mol^−1^ nm^−1^. The system was then equilibrated by a 100 ps simulation conducted under the NVT conditions, followed by an additional 100 ps of simulation under the NPT conditions.

To generate the initial structures for the umbrella sampling, biased MD simulations were conducted. During this process, biasing potentials with a force constant of 1000 kJ mol^−1^ nm^−2^ and a pulling rate of 0.01 nm ps^−1^ were applied along the distance between the COM of the two HBDs. From the resulting trajectory, snapshots were extracted every 10 ps to select representative starting structures for each of the 89 target windows, which spanned the RC range from 0.2 nm to 9.0 nm. Subsequently, for each window, two biased MD simulations were carried out for 200 ns with a restraining force constant of 1000 kJ mol^−1^ nm^−2^ with two randomly and differently selected initial velocities. For equilibration purposes, we discarded the initial 100 ns trajectory from the 200 ns trajectory. Following that, we conducted the PMF calculation and additional analyses. The PMF was computed using WHAM [41] by combining the two sets of trajectories. To estimate statistical uncertainties, the 100–200 ns production phase was partitioned into five 20 ns blocks, and the standard deviation across these blocks was calculated at each point along the reaction coordinate to generate the error bars. For the case where RC was 1.1 nm, an additional 10 trajectories of 200 ns MD simulations were conducted to generate structures for later docking with dimeric aptamers. For 50 ns unbiased MD simulations of VEGF_165_, we used the same protocol as described in the above, starting from the final structures of the biased MD simulations but after removing the biasing potential. For VEGFR-2/VEGF_165_ complex formation, each of the two VEGFR-2 molecules was initially translated 4 nm away from the COM of the dimeric RBDs along the vector connecting this COM and the Cα atom of GLY 220, a residue located at the boundary of the D2 and the D3 subunits of VEGFR-2. The system was then solvated in a 32 nm cubic box using the TIP3P water model, and counter ions were added for neutralization. Then, energy minimization and equilibration MD were performed under the same protocol described for the VEGF_165_ system. Subsequently, the two VEGFR-2 molecules were pulled back in as a rigid body along the same vector toward dimeric RBDs until they reached their original superimposed positions. During this re-associating process, position restraints were applied to the RBDs but HBDs were allowed to relax. A biasing potential with a force constant of 5000 kJ mol^−1^ nm^−2^ and a pulling rate of 0.01 nm ps^−1^ was applied along the pulling direction. For the separate slower pulling simulations performed on selected trajectories (RC ≥ 6.0 nm), the pulling rate was set to 0.001 nm ps^−1^ while keeping the force constant the same. Each resulting VEGFR-2/VEGF_165_ complex was then solvated in a box with a minimum distance of 2 nm between the solute and the box edges. Subsequently, energy minimization and equilibration were conducted under the same conditions as in VEGF_165_, followed by a 100 ns MD simulation.

For each of the 13 different del5-1:del5-1 aptamer homodimers described in the above, 101 binding decoy structures were generated in the following manner. The initially modelled structure was solvated in a box with the minimum distance between the aptamer and the box edge set to 1 nm. TIP3P water was again employed together with counter ions for neutralization. Energy minimization and 500 ns of equilibration were first performed, followed by a sampling simulation of 1000 ns duration. The decoys were sampled at every 10 ns during these sampling simulations.

For the 100 ns MD simulations initiated from the top five docking poses of each of the 13 linker systems—totaling 65 independent trajectories—the same computational protocols as described above were followed. All simulations were conducted under the periodic boundary conditions using GROMACS 2022 [42]. The LINCS algorithm was employed to constrain bonds involving hydrogen atoms, enabling a time step of 2 fs [43]. The short-range Lennard-Jones interactions were cut off at a distance of 1.2 nm, and corrections for long-range dispersion were applied for energy and pressure. The particle mesh Ewald (PME) approach [44] was used for treating the long-range electrostatic interactions with a real-space cutoff of 1.2 nm. The temperature was kept constant at 300 K using the velocity-rescale thermostat [45] with the relaxation time of 1 ps. The pressure was isotropically coupled at 1 bar through the C-rescale barostat [46] with a coupling constant of 1 ps and a compressibility of 4.5 × 10^−5^ bar^−1^. The simulations were conducted on a home-built high-performance computing (HPC) system and relied on Intel 6342 processors together with NVIDIA A100 and A5000 GPUs.

### 3.6. Binding Free Energy Calculations

Binding free energies Δ*G*_bind_ were calculated using the MM-GBSA method as implemented in the gmx_MMPBSA (version 1.6.4) package [47]. The Amber ff19SB force field was applied for the protein, and the OL15 force field was utilized for the DNA aptamer to ensure accurate representation of the complex. For the free energy calculations, snapshots were extracted from the 50–100 ns period of each trajectory at 20 ps intervals. To account for the solvent environment, the Generalized Born (GB) model with the igb = 8 (GB-Neck2) parameter was employed. The ionic strength was set to 0.150 M to simulate physiological salt conditions. Per-residue energy decomposition analysis was performed and entropy contribution was not considered in this study.

## 4. Conclusions

VEGF_165_ is a crucial therapeutic target for angiogenesis-related diseases, with aptamers emerging as promising agents. Despite the enhanced binding affinity of dimeric aptamers over their monomeric counterparts, the inherent flexibility of VEGF_165_ inter-domain linker has hindered the complete structural elucidation of VEGF_165_, posing significant challenges for the rational design of aptamer dimers. To address this challenge of structural flexibility, we calculated the free energy surface shape information as a function of the HBD-HBD distance. The resulting PMF profile showed a distinct energy minimum at 1.1 nm, providing strong evidence for a thermodynamically preferred sandwich-like compact conformation. We next validated the functional relevance of this compact conformation through MD simulations of the VEGFR-2/VEGF_165_ complex. The simulations revealed a clear preference for this type of conformation, and even initially extended conformations tended to transition toward the thermodynamically favored shortened structure. The preference toward the compact structure was rationalized by a hydrogen bond analysis, which confirmed that the compact one forms a more robust interaction network. The hydrogen bonds also matched well with experimentally resolved H-bonds between VEGF_165_ and VEGFR-2.

Although an optimal VEGF_165_ HBD-HBD distance was identified, structural variability was still observed due to the flexible linker, prompting investigation into aptamer homodimer conditions effectively targeting these HBD structures. We constructed del5-1:del5-1 dimers by connecting two units of del5-1 with varying lengths of thymine linkers and docked them to diverse VEGF_165_ structures that were generated still with satisfying the optimal HBD-HBD distance. The results indicated that the binding affinity with VEGF_165_ did not significantly vary with the del5-1:del5-1 linker length. Notably, compact conformations where two HBDs were effectively enveloped by del5-1:del5-1 demonstrated efficient HBD binding. The COM distance between the two del5-1 units was determined to be ~5 nm in general, highlighting the necessity to design an aptamer homodimer that can meet this distance criterion for effective VEGF_165_ binding. These structural and mechanistic insights will enhance our fundamental understanding of VEGF_165_ dynamics and may advance rational designs of aptamers targeting VEGF_165_ toward more effective treatments for angiogenesis-related diseases.

## 5. Limitations

This investigation was conducted entirely in silico. While our simulations identified a thermodynamically preferred compact conformation and specific inter-domain distances, the actual biological environment is far more complex. Factors such as varying pH levels and ionic strengths may influence the protein’s conformational landscape and its binding affinity with aptamers. Therefore, further experimental validation is necessary to confirm the predicted binding affinities and therapeutic potential of the designed aptamer dimers. Despite these limitations, this study may serve as a crucial filtering tool for experimental design. By providing a narrowed range of optimal structural parameters and linker lengths, our findings can offer a theoretical foundation that can significantly reduce the trial-and-error processes in future wet-lab investigations.

## Figures and Tables

**Table 1:** Predicted docking scores and center-of-mass (COM) distances between the two del5-1 aptamer domains in VEGF165/del5-1:del5-1 complexes with varying linker lengths. Values are presented as mean ± standard deviation (SD).

**Linker Length**	**Docking Score ^a,b^**	**Distance (nm) ^c^**
0 dT	−1079.8 ± 74.2	5.6 ± 0.6
1 dT	−1114.4 ± 32.3	5.0 ± 0.2
2 dTs	−1116.0 ± 76.4	5.1 ± 0.4
3 dTs	−1122.5 ± 71.4	4.8 ± 0.3
4 dTs	−1093.2 ± 69.7	4.5 ± 0.4
5 dTs	−1101.7 ± 74.5	5.8 ± 0.4
6 dTs	−1092.6 ± 68.2	5.6 ± 0.3
7 dTs	−1125.2 ± 72.1	5.0 ± 0.3
8 dTs	−1114.5 ± 71.9	5.5 ± 0.2
9 dTs	−1128.0 ± 72.7	5.1 ± 0.1
10 dTs	−1071.6 ± 66.6	5.5 ± 0.2
15 dTs	−1123.2 ± 70.0	5.6 ± 0.3
20 dTs	−1151.6 ± 70.8	4.7 ± 0.3

^a^ In an arbitrary unit. ^b^ Average and SD are from all generated docking poses. ^c^ Average and SD are from top 10 highest-scoring structures.

## Data Availability

The data presented in this study are available in public on FigShare (https://doi.org/10.6084/m9.figshare.30676256 (accessed on 22 November 2025)).

## Supplementary Materials

The following supporting information can be downloaded at: https://www.mdpi.com/article/10.3390/ijms27020712/s1.
