# Supplementary material for: Determining the Optimal Heparin Binding Domain Distance in VEGF165 Using Umbrella Sampling Simulations for Optimal Dimeric Aptamer Design
Source: Int J Mol Sci. 2026 Jan 10;27(2):712. doi: 10.3390/ijms27020712 (PMC12841335; doi:10.3390/ijms27020712)
Supplement: Supplementary file 1 [file ijms-27-00712-s001.zip › LEE_IJMS_SI_final.pdf]

Supplementary Materials for

Determining the optimal heparin binding domain distance in $VEGF_{165}$ using umbrella sampling simulations for optimal dimeric aptamer design

Jung Seok Lee, Yeon Ju Go, and Young Min Rhee*

Department of Chemistry, Korea Advanced Institute of Science and Technology (KAIST), Daejeon 34141, Korea

* E-mail: ymrhee@kaist.ac.kr

Supporting Figures

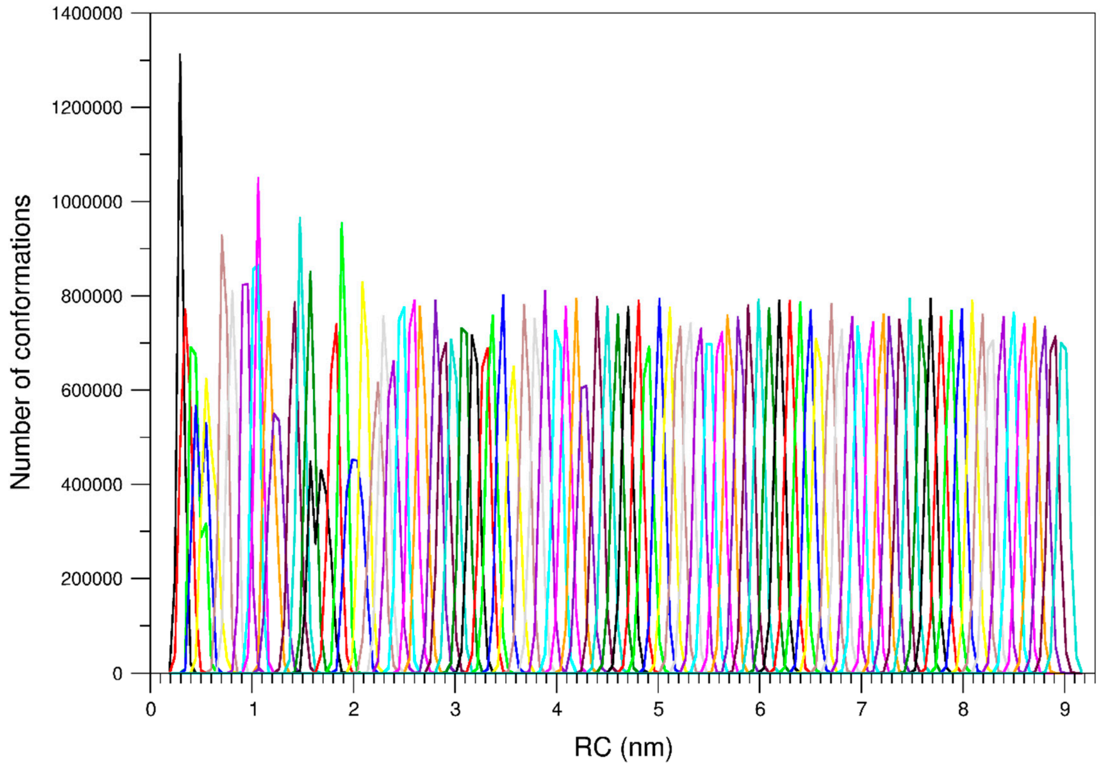

Figure S1. The histogram represents data from 89 umbrella windows, where the RC is defined as the distance between the two HBDs of $VEGF_{165}$. MD simulation trajectories ranging from 100 to 200 ns are utilized to generate the umbrella distributions.

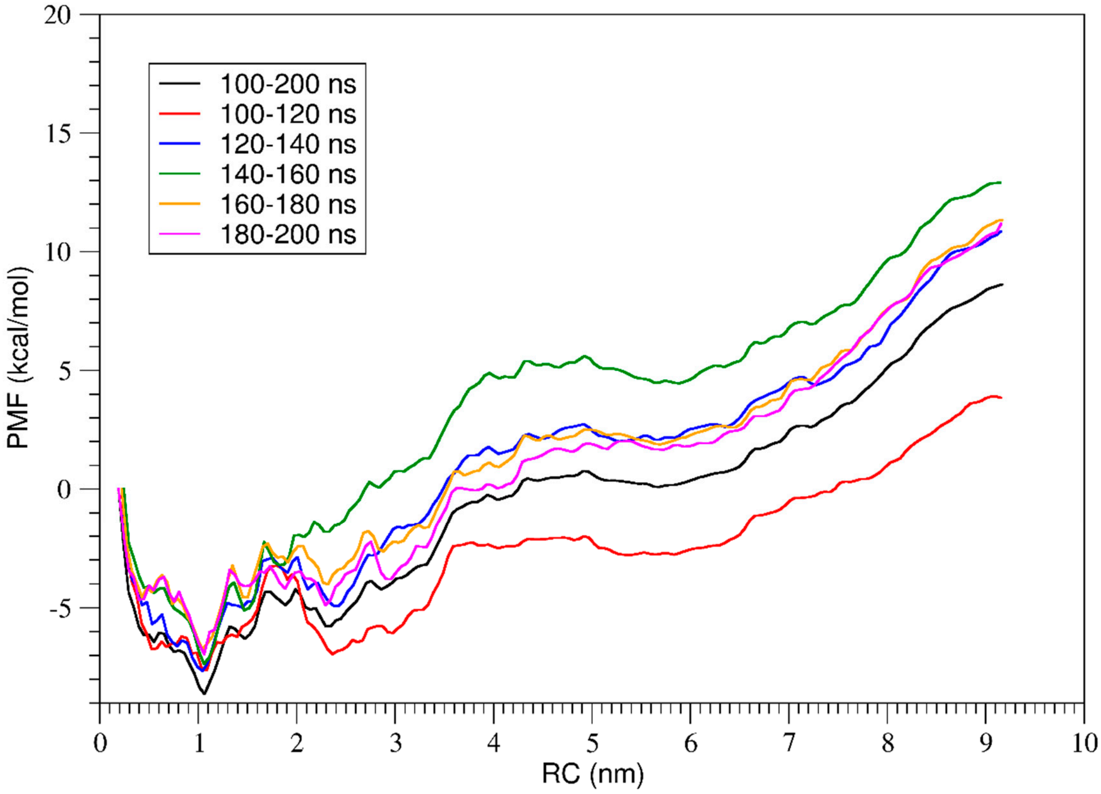

Figure S2. Potential of Mean Force (PMF) profiles partitioned into five blocks. To evaluate the statistical precision and convergence of the free energy calculations, the final 100 ns of the sampling trajectory was divided into five 20 ns blocks.

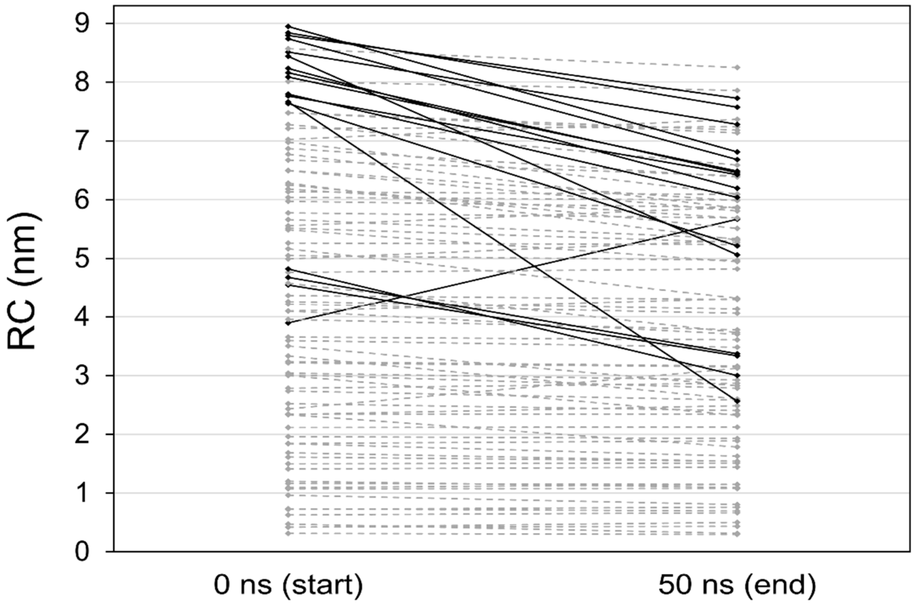

Figure S3. Time evolution of RC during 50 ns of unbiased MD simulations of $VEGF_{165}$ initiated from the final structures of the umbrella sampling simulations. Gray dashed lines represent individual trajectories, while black solid lines highlight trajectories in which RC changed by more than 1 nm over the 50 ns period.

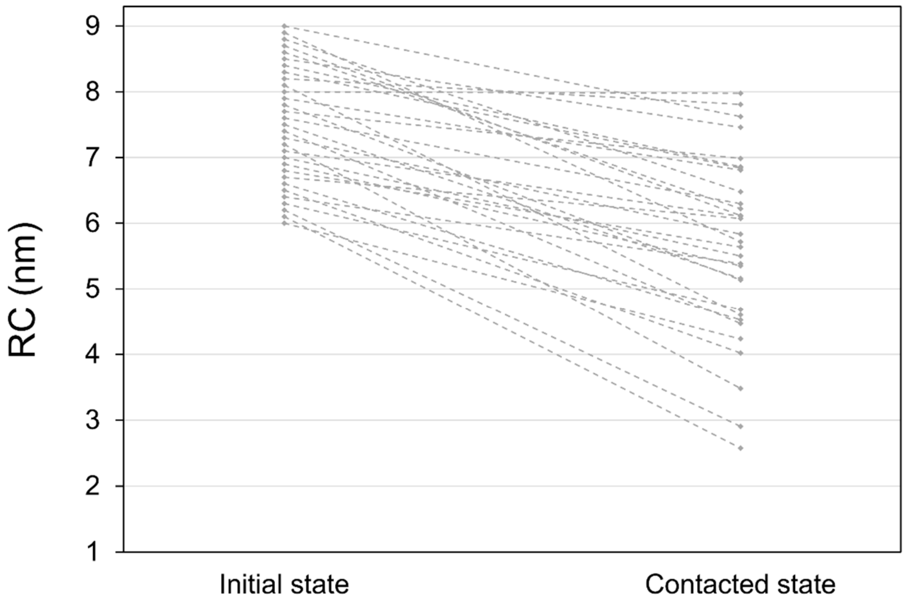

Figure S4. Time evolution of RC during slower pulling simulations of VEGFR-2 toward $VEGF_{165}$ with a pulling rate of 0.001 nm ps^{-1}. To focus on cases that occasionally showed RC increases under the faster pulling protocol in Figure 4 of the main text, only trajectories initiated at RC $\geq$ 6.0 nm were simulated here. Gray dashed lines represent individual trajectories. All trajectories show decreases in RC, indicating that the slower pulling rate allowed longer equilibration time and thus revealed a clearer tendency for $VEGF_{165}$ to compact.

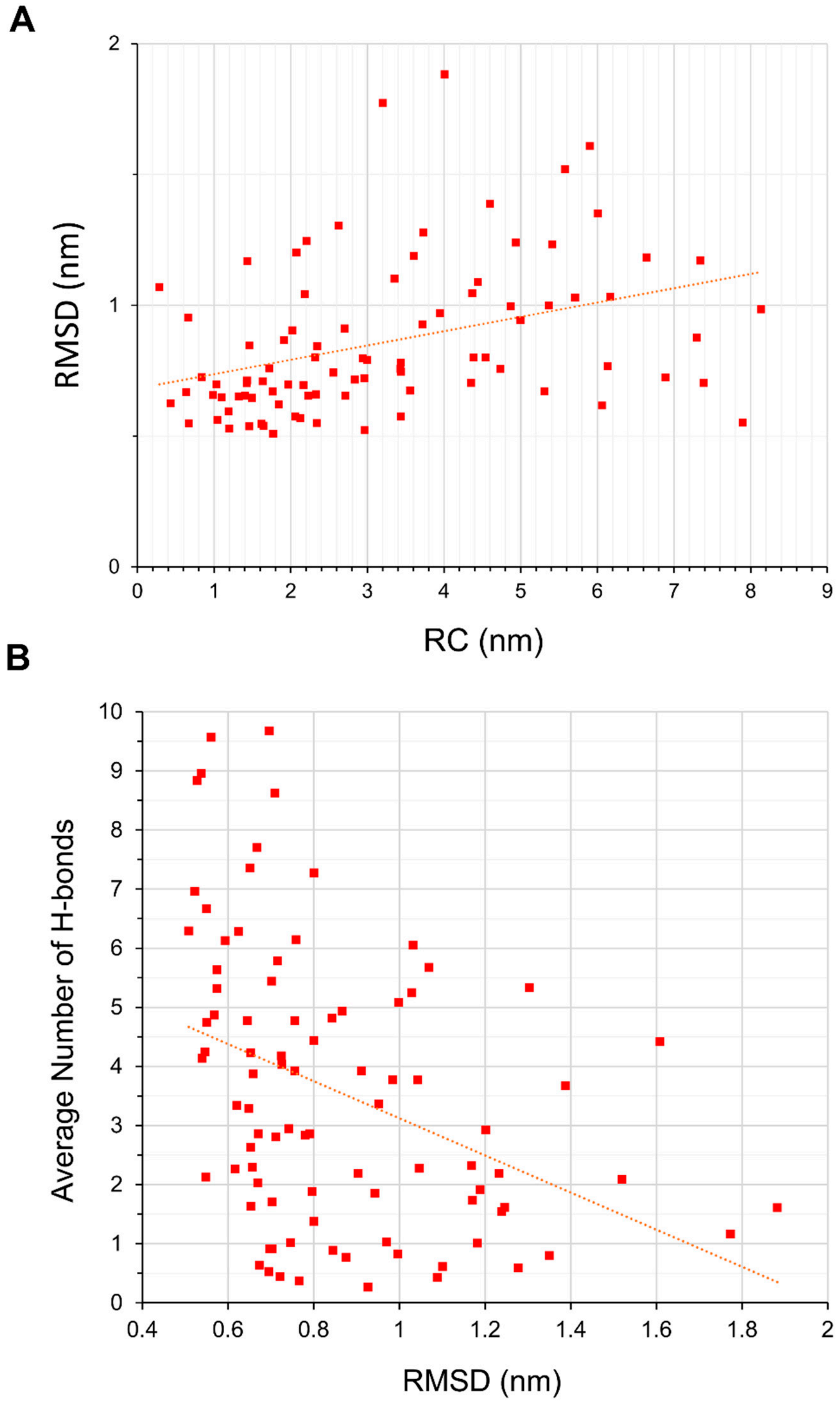

Figure S5. Correlation analysis between VEGFR-2 backbone root-mean-square deviation (RMSD) and binding characteristics, obtained from the $VEGF_{165}$/VEGFR-2 complex simulations. (A) Correlation between VEGFR-2 backbone RMSD and the HBD–HBD distance. (B) Correlation between VEGFR-2 backbone RMSD and the number of hydrogen bonds.

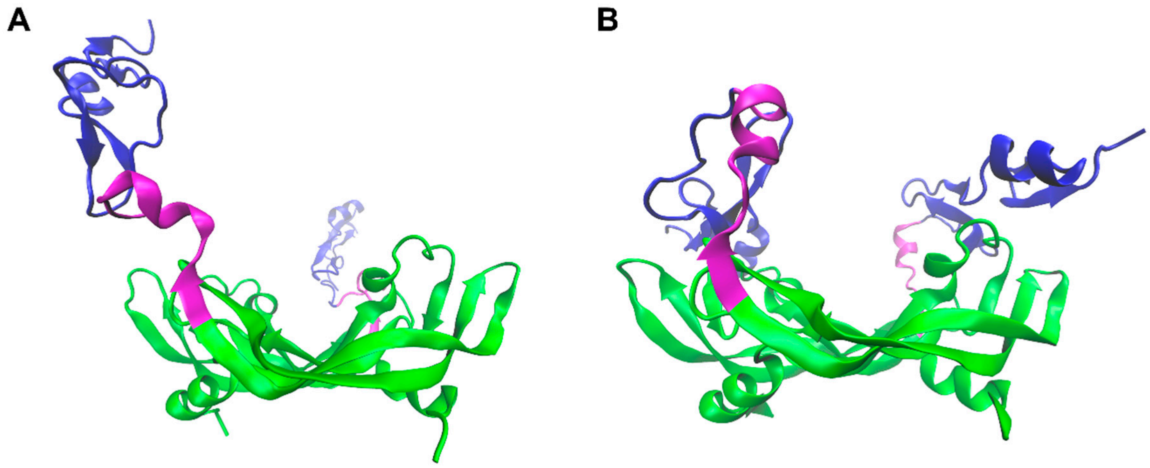

Figure S6. Visual comparison between the (A) constructed $VEGF_{165}$ model and (B) AlphaFold 3 prediction. The overall structures of the individual domains in the AlphaFold 3 predicted structure are similar with the original model used in this study. The observed discrepancy in the HBD–HBD distance is attributed to the intrinsic flexibility of the linker regions, which allows for various spatial orientations of the domains.

Supporting Tables

Table S1. Key hydrogen bonds between VEGFR-2 and $VEGF_{165}$, as identified in the experimental VEGF-2/VEGF-A complex structure.[a]

Bonds no.	Hydrogen bonds	
	VEGFR-2	VEGF-A
1	GLY 196 O	TYR 21 OH
2	TYR 165 O	TYR 25 OH
3	TYR 165 O	ASN 62 ND2
4	TYR 194 O	ASN 62 ND2
5	ASN 253 OD1	GLU 64 N
6	ASN 253 ND2	ASP 63 OD1
7	ASN 253 N	GLU 64 OE1
8	LYS 286 NZ	GLU 64 OE1
9	ILE 256 N	ILE 43 O
10	TYR 137 OH	GLN 89 OE1

[a] Adapted from Brozzo, M. S. et al. [23].

Table S2. Structural stability and thermodynamic binding free energies of the VEGF165–aptamer complexes across 13 linker systems.

Linker length	Pose	ΔG_{bind} (kcal/mol) [a]	Linker–averaged ΔG_{bind} (kcal/mol)	$d_{del1\text{-}HBD}$ (nm) [b]	$d_{del2\text{-}HBD}$ (nm) [b]
T0	pose 1	−149.95 ± 13.47	−152.908	2.26 → 2.18	4.43 → 4.46
	pose 2	−131.76 ± 20.29		4.04 → 2.35	4.83 → 4.97
	pose 3	−145.18 ± 17.29		1.42 → 1.3	5.9 → 5.25
	pose 4	−106.54 ± 11.28		2.3 → 1.99	5.32 → 6.44
	pose 5	−231.11 ± 18.3		2.34 → 1.99	2.87 → 2.59
T1	pose 1	−89.75 ± 10.98	−96.194	5.11 → 5.85	3.03 → 2.92
	pose 2	−51.57 ± 7.4		4.84 → 6.91	2.33 → 2.53
	pose 3	−189.11 ± 20.69		3.27 → 2.74	2.88 → 2.7
	pose 4	−59.09 ± 11.42		4.06 → 3.89	2.99 → 2.81
	pose 5	−91.45 ± 11.01		3.7 → 4.01	3.31 → 3.1
T2	pose 1	−20.21 ± 9.85	−87.11	4.08 → 5.68	4.48 → 5.37
	pose 2	−162.91 ± 16.27		1.56 → 1.38	4.78 → 4.29
	pose 3	−75.08 ± 12.85		3.57 → 3.63	4.55 → 5.67
	pose 4	−86.35 ± 19.52		4.73 → 4.81	3.16 → 3.15
	pose 5	−91.0 ± 13.1		3.77 → 4.44	3.11 → 2.61
T3	pose 1	−107.66 ± 12.08	−100.4	3.46 → 4.15	2.96 → 2.7
	pose 2	−119.54 ± 17.3		3.38 → 3.57	1.86 → 1.9
	pose 3	−87.96 ± 11.0		2.49 → 2.64	4.47 → 4.55
	pose 4	−80.92 ± 13.59		4.29 → 3.68	2.61 → 2.57

	pose 5	−105.92 ± 12.7		2.94 → 2.93	4.88 → 5.44
T4	pose 1	−128.67 ± 15.31	−100.942	2.56 → 2.99	2.39 → 2.61
	pose 2	−132.83 ± 13.07		2.25 → 2.29	3.74 → 3.37
	pose 3	−116.1 ± 14.96		3.31 → 3.24	2.57 → 2.77
	pose 4	−84.9 ± 14.29		3.18 → 2.64	4.57 → 4.69
	pose 5	−42.21 ± 11.34		4.97 → 6.89	3.09 → 3.01
T5	pose 1	−176.4 ± 16.18	−127.322	2.68 → 2.68	4.23 → 3.81
	pose 2	−178.91 ± 18.44		4.78 → 5.06	1.9 → 1.78
	pose 3	−86.04 ± 21.01		4.28 → 5.75	2.69 → 2.67
	pose 4	−84.21 ± 13.66		1.99 → 2.33	5.14 → 5.3
	pose 5	−111.05 ± 13.78		3.75 → 3.76	2.44 → 2.4
T6	pose 1	−195.49 ± 16.37	−149.902	3.73 → 3.45	2.41 → 2.4
	pose 2	−151.82 ± 21.24		4.45 → 4.8	2.44 → 1.93
	pose 3	−130.36 ± 16.53		2.77 → 2.85	4.15 → 3.99
	pose 4	−124.5 ± 16.94		2.8 → 2.83	3.81 → 4.3
	pose 5	−147.34 ± 24.93		2.65 → 2.96	4.48 → 4.14
T7	pose 1	−146.47 ± 15.87	−105.07	1.91 → 2.01	3.69 → 3.58
	pose 2	−87.1 ± 12.8		4.49 → 4.6	3.86 → 3.94
	pose 3	−127.69 ± 19.76		3.77 → 4.29	2.96 → 2.52
	pose 4	−16.13 ± 9.77		3.94 → 4.0	4.53 → 4.85
	pose 5	−147.96 ± 13.99		3.72 → 3.57	2.71 → 2.73
T8	pose 1	−149.59 ± 13.44	−80.41	3.21 → 3.23	3.69 → 3.43
	pose 2	−48.97 ± 9.82		5.92 → 7.2	3.98 → 3.77

	pose 3	−80.21 ± 14.4		2.99 → 3.28	4.34 → 5.18
	pose 4	−21.39 ± 8.97		4.79 → 4.78	4.57 → 5.1
	pose 5	−101.89 ± 16.73		4.44 → 4.06	4.31 → 4.75
T9	pose 1	−157.07 ± 15.98	−101.428	3.16 → 3.26	3.19 → 3.03
	pose 2	−86.6 ± 14.48		3.42 → 3.49	4.05 → 3.98
	pose 3	−71.82 ± 12.76		4.45 → 5.42	4.66 → 3.18
	pose 4	−106.57 ± 12.7		2.43 → 2.92	4.9 → 5.04
	pose 5	−85.08 ± 13.67		4.04 → 3.27	3.55 → 3.64
T10	pose 1	−119.05 ± 12.34	−102.096	5.15 → 5.26	2.19 → 1.86
	pose 2	−105.3 ± 13.91		4.12 → 4.27	3.03 → 2.96
	pose 3	−138.18 ± 16.23		5.28 → 5.01	2.42 → 2.67
	pose 4	−50.44 ± 10.06		4.56 → 4.75	5.63 → 6.15
	pose 5	−97.51 ± 11.81		4.17 → 4.26	3.19 → 3.35
T15	pose 1	−121.9 ± 20.79	−117.334	2.43 → 2.3	4.03 → 4.05
	pose 2	−124.1 ± 17.25		6.04 → 4.79	1.87 → 1.89
	pose 3	−133.54 ± 14.24		2.81 → 2.64	4.09 → 3.74
	pose 4	−74.47 ± 13.33		1.69 → 1.99	5.04 → 4.47
	pose 5	−132.66 ± 21.04		4.17 → 4.03	3.15 → 2.91
T20	pose 1	−136.64 ± 15.84	−145.45	3.87 → 3.89	2.98 → 2.89
	pose 2	−126.03 ± 16.64		2.36 → 2.24	4.71 → 4.13
	pose 3	−176.9 ± 16.56		3.12 → 3.0	5.8 → 5.64
	pose 4	−137.84 ± 34.49		3.01 → 3.21	2.91 → 2.78
	pose 5	−149.84 ± 17.76		3.92 → 4.0	2.89 → 2.69

[a] Uncertainties are presented with statistical standard deviations.

[b] $d_{del1\text{-}HBD}$ and $d_{del2\text{-}HBD}$ denote the center-of-mass distances between the $VEGF_{165}$ HBD and each of the two del5-1 aptamer domains, respectively. Values represent the distance in the initial docking state followed by the mean distance calculated during the 50–100 ns production phase.
