# Supplementary figures and images for: Determining the Optimal Heparin Binding Domain Distance in VEGF165 Using Umbrella Sampling Simulations for Optimal Dimeric Aptamer Design
Source: Int J Mol Sci. 2026 Jan 10;27(2):712. doi: 10.3390/ijms27020712 (PMC12841335; doi:10.3390/ijms27020712)

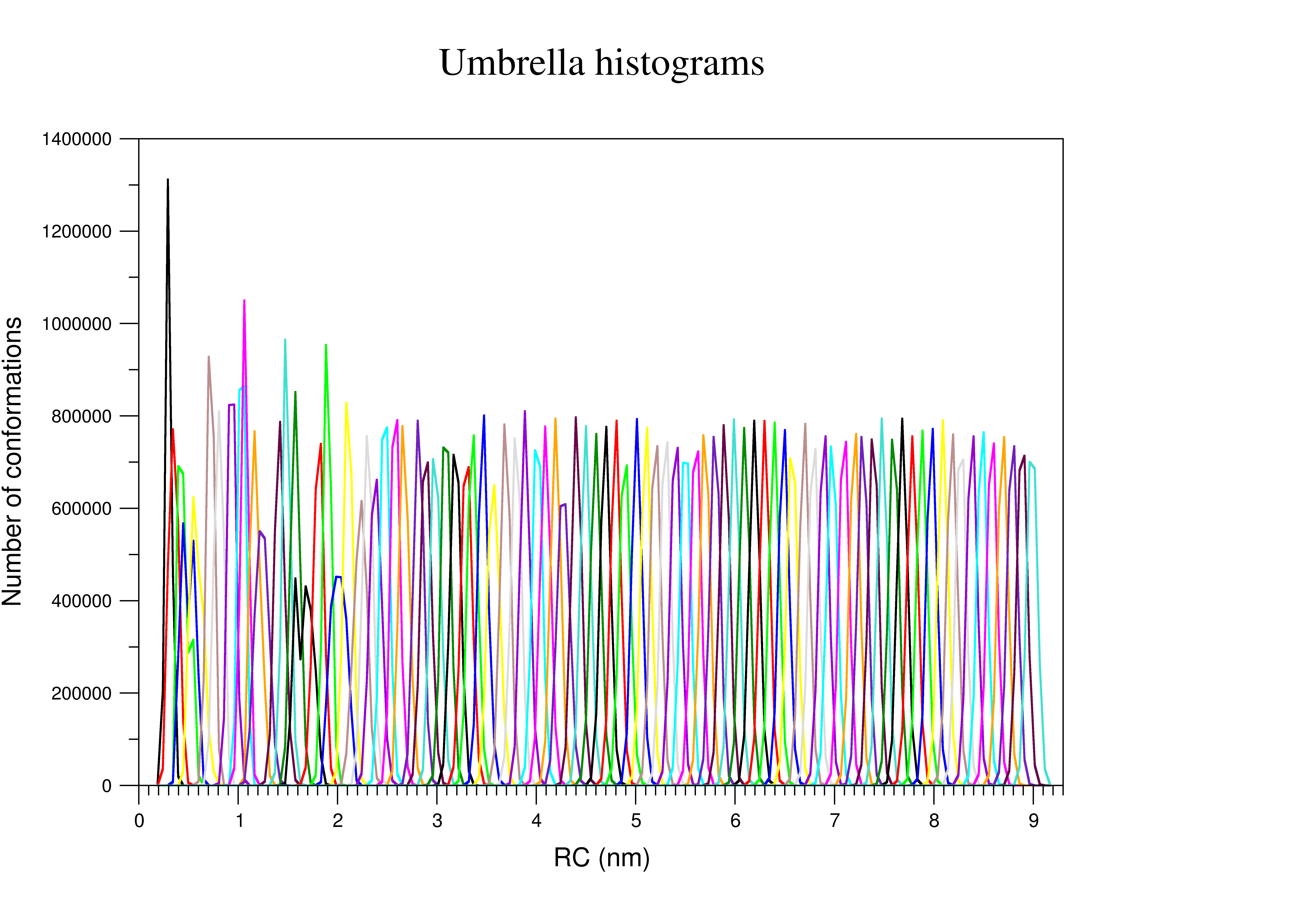

Supplement: Supplementary file 1 [file ijms-27-00712-s001.zip › Figure S1.png]

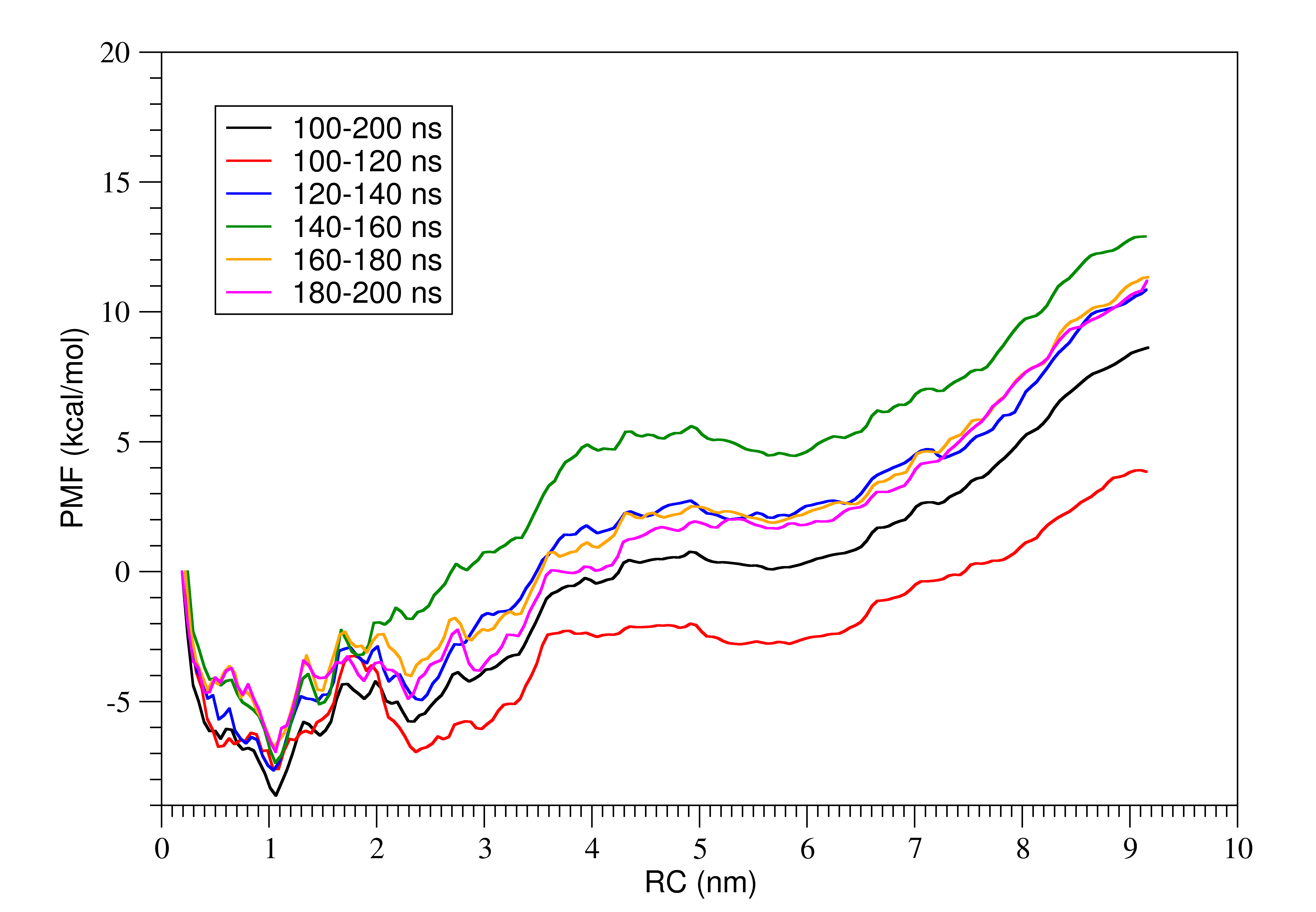

Supplement: Supplementary file 1 [file ijms-27-00712-s001.zip › Figure S2.png]

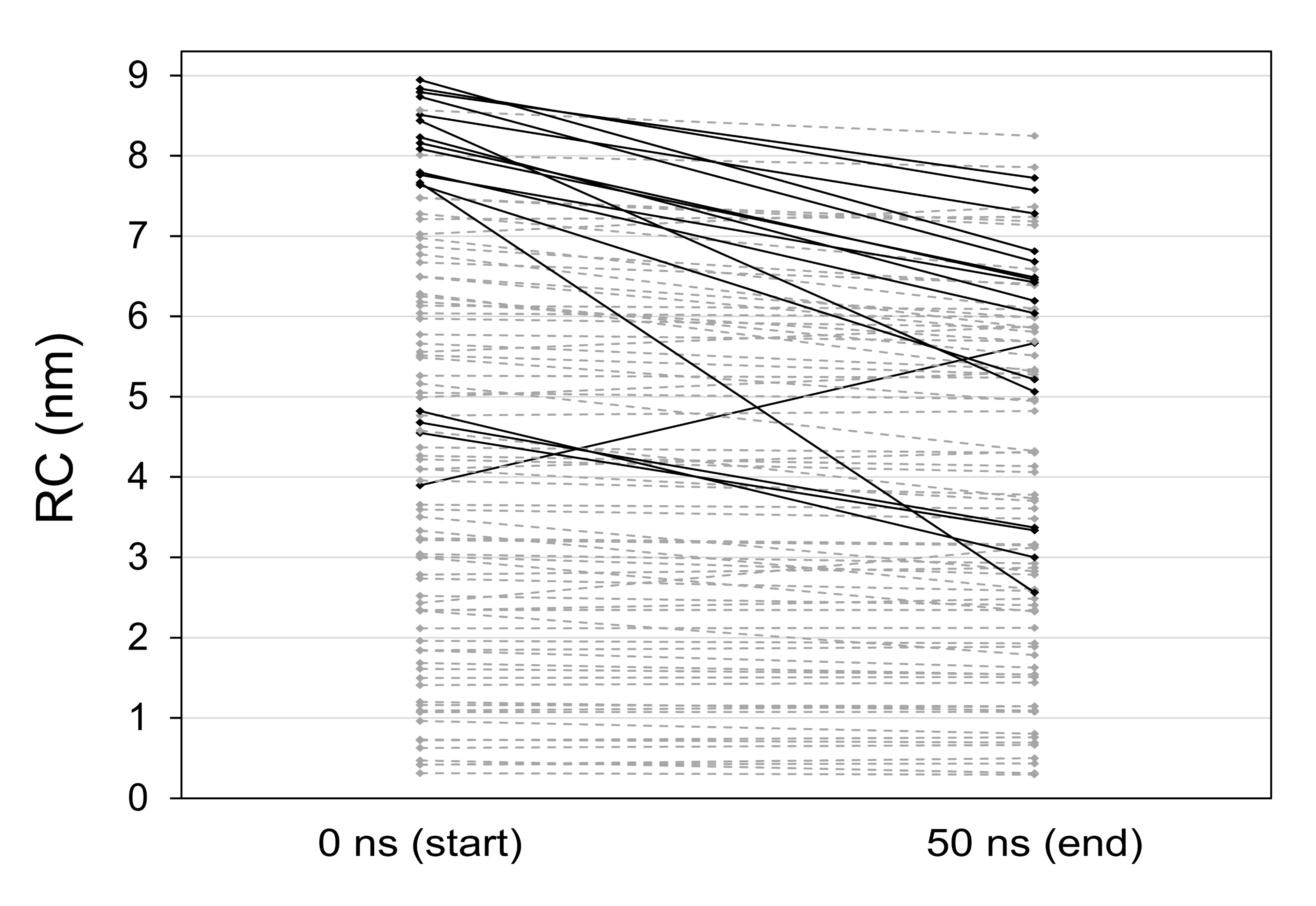

Supplement: Supplementary file 1 [file ijms-27-00712-s001.zip › Figure S3.tif]

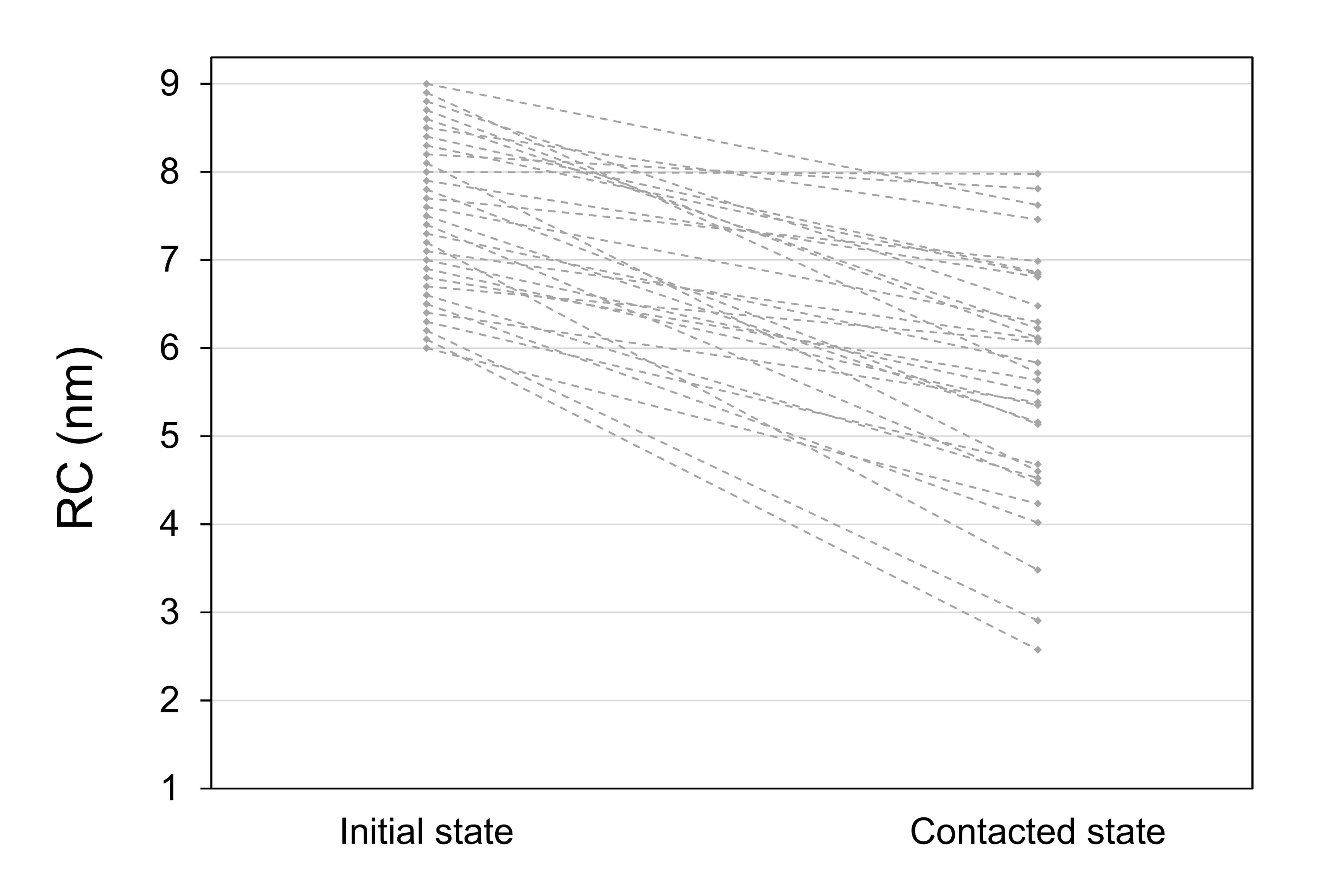

Supplement: Supplementary file 1 [file ijms-27-00712-s001.zip › Figure S4.tif]

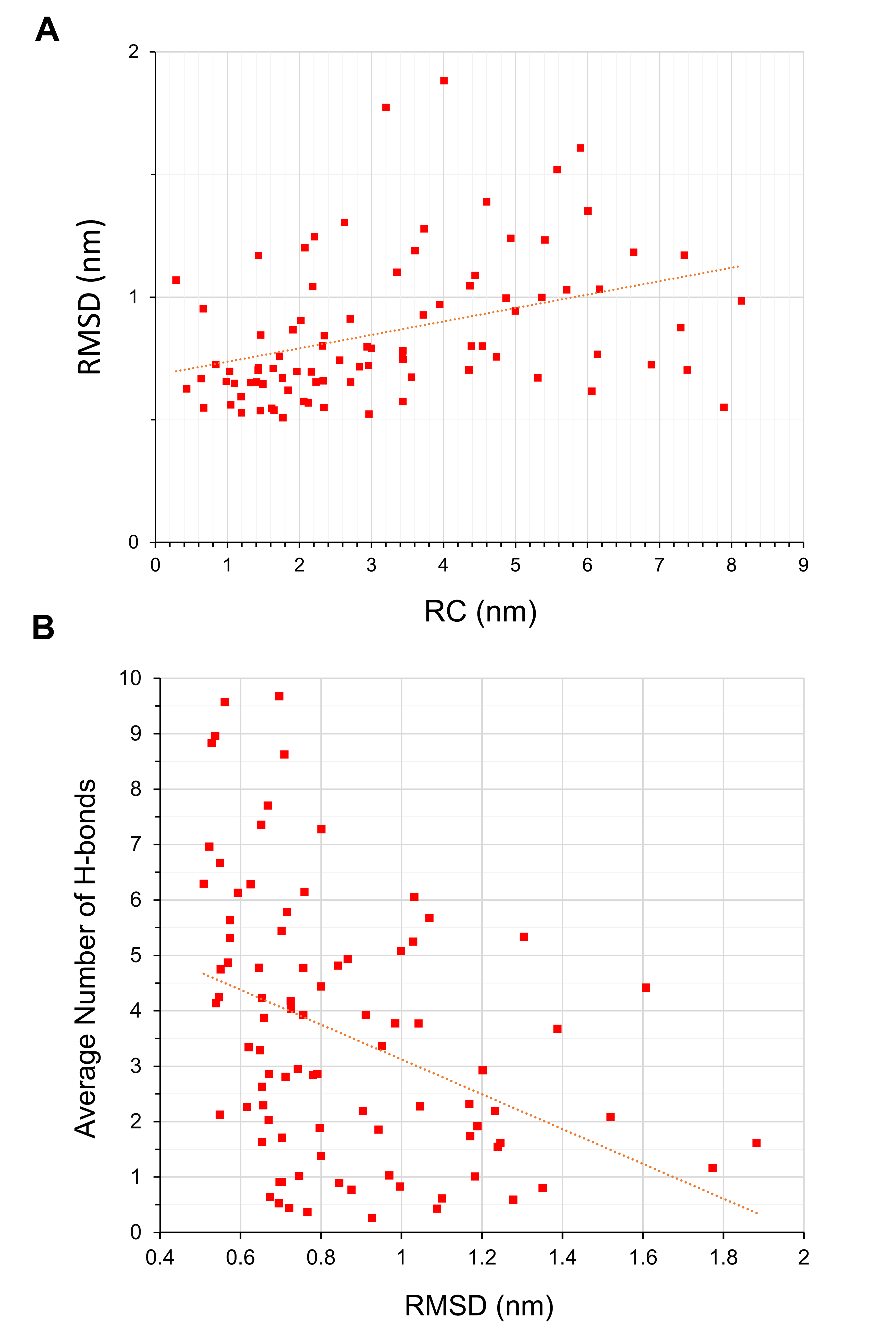

Supplement: Supplementary file 1 [file ijms-27-00712-s001.zip › Figure S5.tif]

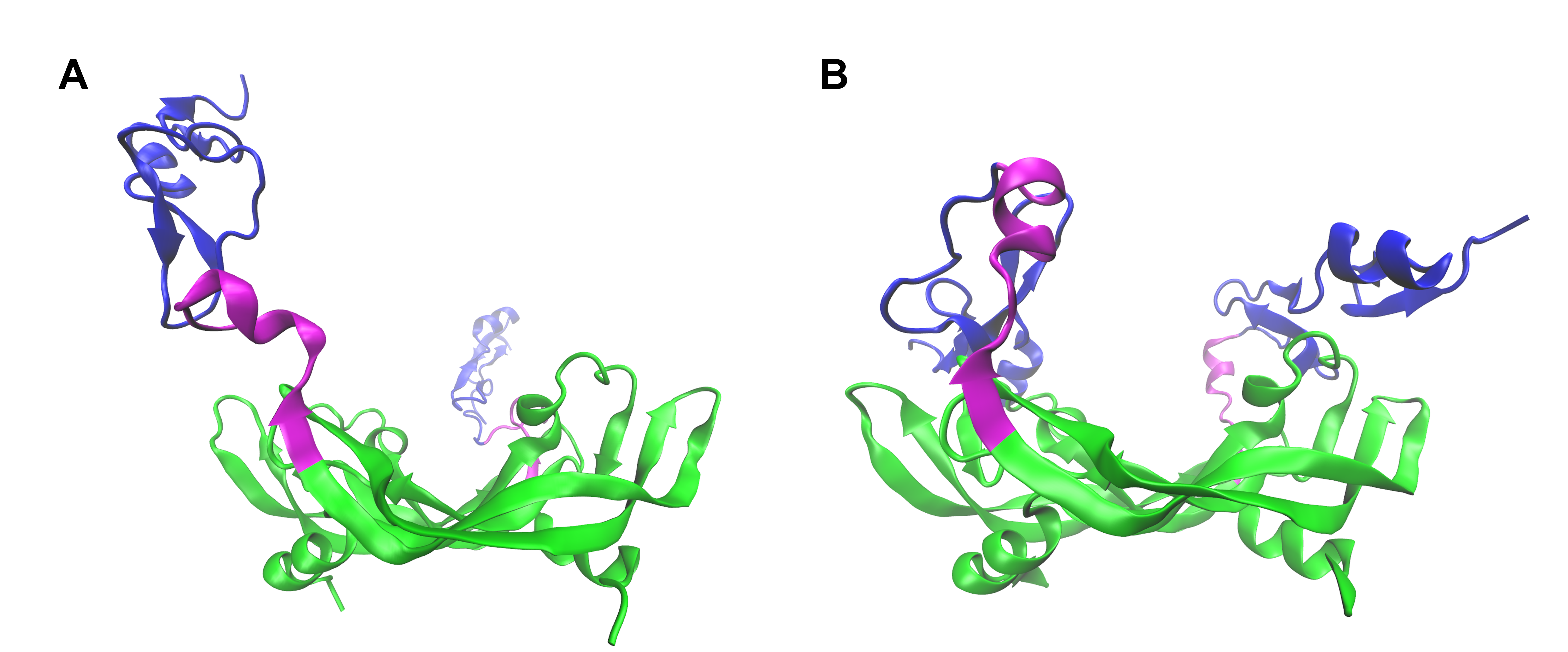

Supplement: Supplementary file 1 [file ijms-27-00712-s001.zip › Figure S6.tif]
